# *catena*-Poly[[dichloridocopper(II)]-μ-4,4′-bis­(benzimidazol-1-yl)biphen­yl]

**DOI:** 10.1107/S1600536811011421

**Published:** 2011-04-07

**Authors:** Dan-Ni Xiao, Hong-Yan Pan, Min Yao, Gang Xie

**Affiliations:** a Key Laboratory of Synthetic and Natural Functional Molecule Chemistry (Ministry of Education), College of Chemistry & Materials Science, Northwest University, Xi’an 710069, People’s Republic of China; b The College of Life Sciences, Northwest University, Xi’an 710069, People’s Republic of China

## Abstract

In the title compound, [CuCl_2_(C_26_H_18_N_4_)]_*n*_, the Cu(II) ion is four-coordinated by two N atoms from two 4,4′-bis­(benzo­imidazol-1-yl)biphenyl ligands and two chloride anions, in a slightly distorted tetra­hedral environment. The biphenyl ligand acts as a linear bidentate ligand, connecting the metal atoms into an infinite chain parallel to [101]. In the biphenyl ligand, the two benzene rings make a dihedral angle of 33.19 (7)°.

## Related literature

For background to benzimidazole-based ligands in crystal engineering, see: Jin *et al.* (2006 ▶); Li *et al.* (2009 ▶); Su *et al.* (2003 ▶).
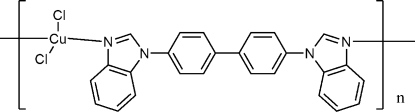


## Experimental

### 

#### Crystal data

[CuCl_2_(C_26_H_18_N_4_)]
*M* *_r_* = 520.88
Monoclinic, 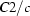

*a* = 12.599 (4) Å
*b* = 15.280 (4) Å
*c* = 11.233 (3) Å
β = 91.936 (4)°
*V* = 2161.3 (10) Å^3^
*Z* = 4
Mo *K*α radiation
μ = 1.28 mm^−1^
*T* = 293 K
0.04 × 0.03 × 0.02 mm

#### Data collection

Rigaku Mercury CCD area-detector diffractometer
Absorption correction: multi-scan (*CrystalClear*; Rigaku/MSC, 2005 ▶) *T*_min_ = 0.955, *T*_max_ = 0.975
6675 measured reflections
1903 independent reflections
1761 reflections with *I* > 2σ(*I*)
*R*_int_ = 0.027

#### Refinement

*R*[*F* ^2^ > 2σ(*F* ^2^)] = 0.026
*wR*(*F* ^2^) = 0.066
*S* = 1.06
1903 reflections
151 parameters
H-atom parameters constrained
Δρ_max_ = 0.50 e Å^−3^
Δρ_min_ = −0.27 e Å^−3^

### 

Data collection: *CrystalClear* (Rigaku/MSC, 2005 ▶); cell refinement: *CrystalClear*; data reduction: *CrystalClear*; program(s) used to solve structure: *SHELXS97* (Sheldrick, 2008 ▶); program(s) used to refine structure: *SHELXL97* (Sheldrick, 2008 ▶); molecular graphics: *SHELXTL* (Sheldrick, 2008 ▶); software used to prepare material for publication: *SHELXTL*.

## Supplementary Material

Crystal structure: contains datablocks I, global. DOI: 10.1107/S1600536811011421/fj2406sup1.cif

Structure factors: contains datablocks I. DOI: 10.1107/S1600536811011421/fj2406Isup2.hkl

Additional supplementary materials:  crystallographic information; 3D view; checkCIF report

## Figures and Tables

**Fig. 1.**
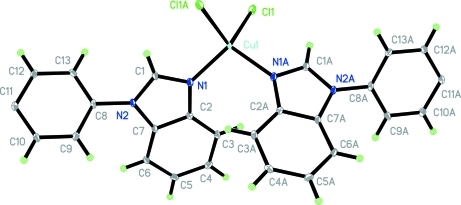
The molecular structure of (I). Displacement ellipsoids are drawn at the 30% probability level and H atoms are shown as small spheres of arbitrary radius.

**Fig. 2.**
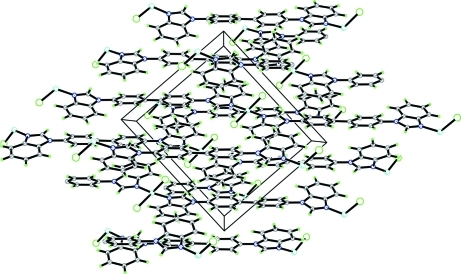
The crystal packing for (I).

## supplementary crystallographic information

### Comment

Benzoimidazole has been well used in crystal engineering, and a large number of benzoimidazole-containing flexible ligands have been extensively studied (Su *et al.*,2003; Jin *et al.*,2006). However, to our knowledge, the research on benzoimidazole ligands bearing rigid spacers is still less developed (Li *et al.*,2009).

Single-crystal X-ray diffraction analysis reveals that the title compound (I) crystallizes in the monoclinic space group *C2/c*. The geometry of the Cu(II) ion is surrounded by two benzoiimidazole rings of distinct L ligands and two chlorine anions, which illustrates a slightly distorted tetrahedral coordination environment (Fig. 1). Notably, as shown in Fig. 2, the four-coordinated Cu(II) center is bridged by the linear ligand L to form an infinite one-dimensional architecture.

### Experimental

A mixture of CH_3_OH and CHCl_3_ (1:1, 8 ml), as a buffer layer, was carefully layered over a solution of 4,4'-Bis(benzoimidazol-1-yl)terphenyl (L, 0.06 mmol) in CHCl_3_ (6 ml). Then a solution of CuCl_2_ (0.06 mmol) in CH_3_OH (6 ml) was layered over the buffer layer, and the resultant reaction was left to stand at room temperature. After *ca* three weeks, purple block single crystals appeared at the boundary. Yield: ~35% (based on L).

### Refinement

C-bound H atoms were positioned geometrically and refined in the riding-model approximation, with C—H = 0.93Å and *U*_iso_(H) = 1.2Ueq.

### Crystal data

**Table:**

[CuCl_2_(C_26_H_18_N_4_)]	*F*(000) = 1060
*M**_r_* = 520.88	*D*_x_ = 1.601 Mg m^−^^3^
Monoclinic, *C*2/*c*	Mo *K*α radiation, λ = 0.71073 Å
Hall symbol: -C 2yc	Cell parameters from 2903 reflections
*a* = 12.599 (4) Å	θ = 2.1–27.9°
*b* = 15.280 (4) Å	µ = 1.28 mm^−^^1^
*c* = 11.233 (3) Å	*T* = 293 K
β = 91.936 (4)°	Block, brown
*V* = 2161.3 (10) Å^3^	0.04 × 0.03 × 0.02 mm
*Z* = 4	

### Data collection

**Table:**

Rigaku Mercury CCD area-detector diffractometer	1903 independent reflections
Radiation source: fine-focus sealed tube	1761 reflections with *I* > 2σ(*I*)
graphite	*R*_int_ = 0.027
Detector resolution: 9 pixels mm^-1^	θ_max_ = 25.0°, θ_min_ = 3.2°
ω scans	*h* = −14→14
Absorption correction: multi-scan (*CrystalClear*; Rigaku/MSC, 2005)	*k* = −18→18
*T*_min_ = 0.955, *T*_max_ = 0.975	*l* = −13→13
6675 measured reflections	

### Refinement

**Table:**

Refinement on *F*^2^	Primary atom site location: structure-invariant direct methods
Least-squares matrix: full	Secondary atom site location: difference Fourier map
*R*[*F*^2^ > 2σ(*F*^2^)] = 0.026	Hydrogen site location: inferred from neighbouring sites
*wR*(*F*^2^) = 0.066	H-atom parameters constrained
*S* = 1.06	*w* = 1/[σ^2^(*F*_o_^2^) + (0.0296*P*)^2^ + 3.937*P*] where *P* = (*F*_o_^2^ + 2*F*_c_^2^)/3
1903 reflections	(Δ/σ)_max_ = 0.002
151 parameters	Δρ_max_ = 0.50 e Å^−^^3^
0 restraints	Δρ_min_ = −0.27 e Å^−^^3^

### Special details

**Table:**

Geometry. All e.s.d.'s (except the e.s.d. in the dihedral angle between two l.s. planes) are estimated using the full covariance matrix. The cell e.s.d.'s are taken into account individually in the estimation of e.s.d.'s in distances, angles and torsion angles; correlations between e.s.d.'s in cell parameters are only used when they are defined by crystal symmetry. An approximate (isotropic) treatment of cell e.s.d.'s is used for estimating e.s.d.'s involving l.s. planes.
Refinement. Refinement of *F*^2^ against ALL reflections. The weighted *R*-factor *wR* and goodness of fit *S* are based on *F*^2^, conventional *R*-factors *R* are based on *F*, with *F* set to zero for negative *F*^2^. The threshold expression of *F*^2^ > σ(*F*^2^) is used only for calculating *R*-factors(gt) *etc*. and is not relevant to the choice of reflections for refinement. *R*-factors based on *F*^2^ are statistically about twice as large as those based on *F*, and *R*- factors based on ALL data will be even larger.

### Fractional atomic coordinates and isotropic or equivalent isotropic displacement parameters (Å^2^)

**Table:**

	*x*	*y*	*z*	*U*_iso_*/*U*_eq_	
Cu1	1.0000	1.09184 (2)	0.7500	0.01275 (12)	
Cl1	0.98903 (4)	1.18252 (3)	0.59365 (5)	0.02221 (15)	
N1	0.89293 (14)	1.01051 (11)	0.81368 (14)	0.0136 (4)	
N2	0.75910 (13)	0.96997 (11)	0.92601 (15)	0.0128 (4)	
C1	0.83320 (16)	1.03097 (13)	0.90401 (18)	0.0138 (4)	
H1	0.8414	1.0822	0.9480	0.017*	
C2	0.85403 (16)	0.92994 (13)	0.77153 (18)	0.0121 (4)	
C3	0.88572 (16)	0.87841 (13)	0.67670 (17)	0.0133 (4)	
H3	0.9422	0.8949	0.6304	0.016*	
C4	0.83018 (17)	0.80212 (13)	0.65424 (19)	0.0158 (4)	
H4	0.8493	0.7666	0.5913	0.019*	
C5	0.74523 (17)	0.77700 (13)	0.72469 (19)	0.0168 (5)	
H5	0.7089	0.7255	0.7064	0.020*	
C6	0.71401 (17)	0.82661 (13)	0.82038 (19)	0.0142 (4)	
H6	0.6584	0.8095	0.8676	0.017*	
C7	0.77056 (16)	0.90357 (13)	0.84203 (17)	0.0125 (4)	
C8	0.68519 (16)	0.97360 (13)	1.02045 (18)	0.0132 (4)	
C9	0.66932 (17)	0.89992 (14)	1.08940 (19)	0.0177 (5)	
H9	0.7071	0.8488	1.0756	0.021*	
C10	0.59681 (17)	0.90309 (14)	1.17892 (19)	0.0177 (5)	
H10	0.5857	0.8533	1.2245	0.021*	
C11	0.53980 (16)	0.97924 (13)	1.20262 (17)	0.0129 (4)	
C12	0.55967 (16)	1.05353 (13)	1.13412 (18)	0.0129 (4)	
H12	0.5247	1.1056	1.1502	0.015*	
C13	0.63069 (16)	1.05066 (13)	1.04257 (18)	0.0137 (4)	
H13	0.6418	1.1000	0.9962	0.016*	

### Atomic displacement parameters (Å^2^)

**Table:**

	*U*^11^	*U*^22^	*U*^33^	*U*^12^	*U*^13^	*U*^23^
Cu1	0.0123 (2)	0.01272 (19)	0.01364 (19)	0.000	0.00700 (14)	0.000
Cl1	0.0252 (3)	0.0219 (3)	0.0202 (3)	0.0108 (2)	0.0105 (2)	0.0070 (2)
N1	0.0132 (9)	0.0158 (9)	0.0121 (9)	−0.0018 (7)	0.0043 (7)	−0.0008 (7)
N2	0.0121 (9)	0.0151 (8)	0.0114 (8)	−0.0016 (7)	0.0052 (7)	0.0000 (7)
C1	0.0118 (11)	0.0166 (10)	0.0131 (10)	−0.0021 (8)	0.0037 (8)	−0.0004 (8)
C2	0.0110 (10)	0.0138 (10)	0.0114 (10)	−0.0011 (8)	0.0011 (8)	0.0014 (8)
C3	0.0119 (11)	0.0172 (10)	0.0108 (10)	0.0016 (8)	0.0022 (8)	0.0018 (8)
C4	0.0178 (11)	0.0155 (10)	0.0141 (10)	0.0043 (8)	0.0010 (9)	−0.0024 (8)
C5	0.0164 (11)	0.0115 (10)	0.0226 (11)	−0.0030 (8)	0.0003 (9)	0.0013 (9)
C6	0.0112 (10)	0.0140 (10)	0.0177 (11)	−0.0012 (8)	0.0035 (8)	0.0036 (8)
C7	0.0106 (10)	0.0155 (10)	0.0114 (10)	0.0011 (8)	0.0031 (8)	0.0018 (8)
C8	0.0100 (10)	0.0198 (11)	0.0099 (10)	−0.0020 (8)	0.0033 (8)	−0.0003 (8)
C9	0.0183 (12)	0.0173 (11)	0.0181 (11)	0.0054 (9)	0.0082 (9)	0.0019 (9)
C10	0.0197 (12)	0.0180 (11)	0.0160 (11)	0.0011 (9)	0.0082 (9)	0.0055 (9)
C11	0.0101 (11)	0.0175 (11)	0.0112 (10)	−0.0003 (8)	0.0018 (8)	−0.0009 (8)
C12	0.0103 (10)	0.0141 (10)	0.0142 (10)	−0.0003 (8)	0.0015 (8)	−0.0028 (8)
C13	0.0146 (11)	0.0135 (10)	0.0130 (10)	−0.0045 (8)	0.0018 (8)	0.0016 (8)

### Geometric parameters (Å, °)

**Table:**

Cu1—N1^i^	1.9851 (17)	C5—C6	1.383 (3)
Cu1—N1	1.9851 (17)	C5—H5	0.9300
Cu1—Cl1^i^	2.2378 (7)	C6—C7	1.392 (3)
Cu1—Cl1	2.2378 (7)	C6—H6	0.9300
N1—C1	1.321 (3)	C8—C9	1.385 (3)
N1—C2	1.402 (3)	C8—C13	1.390 (3)
N2—C1	1.348 (3)	C9—C10	1.382 (3)
N2—C7	1.396 (3)	C9—H9	0.9300
N2—C8	1.436 (3)	C10—C11	1.398 (3)
C1—H1	0.9300	C10—H10	0.9300
C2—C3	1.394 (3)	C11—C12	1.399 (3)
C2—C7	1.397 (3)	C11—C11^ii^	1.487 (4)
C3—C4	1.379 (3)	C12—C13	1.387 (3)
C3—H3	0.9300	C12—H12	0.9300
C4—C5	1.406 (3)	C13—H13	0.9300
C4—H4	0.9300		
			
N1^i^—Cu1—N1	102.49 (10)	C4—C5—H5	119.0
N1^i^—Cu1—Cl1^i^	130.22 (5)	C5—C6—C7	116.19 (19)
N1—Cu1—Cl1^i^	97.44 (5)	C5—C6—H6	121.9
N1^i^—Cu1—Cl1	97.44 (5)	C7—C6—H6	121.9
N1—Cu1—Cl1	130.22 (5)	C6—C7—N2	131.90 (19)
Cl1^i^—Cu1—Cl1	103.48 (4)	C6—C7—C2	122.36 (19)
C1—N1—C2	105.40 (17)	N2—C7—C2	105.69 (17)
C1—N1—Cu1	122.71 (14)	C9—C8—C13	120.48 (19)
C2—N1—Cu1	131.47 (14)	C9—C8—N2	119.42 (18)
C1—N2—C7	106.89 (17)	C13—C8—N2	120.10 (18)
C1—N2—C8	125.39 (17)	C10—C9—C8	119.3 (2)
C7—N2—C8	127.71 (17)	C10—C9—H9	120.3
N1—C1—N2	113.16 (18)	C8—C9—H9	120.3
N1—C1—H1	123.4	C9—C10—C11	121.6 (2)
N2—C1—H1	123.4	C9—C10—H10	119.2
C3—C2—C7	120.72 (18)	C11—C10—H10	119.2
C3—C2—N1	130.42 (19)	C10—C11—C12	117.91 (19)
C7—C2—N1	108.85 (18)	C10—C11—C11^ii^	119.99 (13)
C4—C3—C2	117.44 (19)	C12—C11—C11^ii^	122.10 (13)
C4—C3—H3	121.3	C13—C12—C11	120.95 (19)
C2—C3—H3	121.3	C13—C12—H12	119.5
C3—C4—C5	121.28 (19)	C11—C12—H12	119.5
C3—C4—H4	119.4	C12—C13—C8	119.64 (19)
C5—C4—H4	119.4	C12—C13—H13	120.2
C6—C5—C4	121.99 (19)	C8—C13—H13	120.2
C6—C5—H5	119.0		
			
N1^i^—Cu1—N1—C1	151.17 (19)	C8—N2—C7—C6	3.6 (3)
Cl1^i^—Cu1—N1—C1	17.06 (16)	C1—N2—C7—C2	−0.2 (2)
Cl1—Cu1—N1—C1	−97.67 (16)	C8—N2—C7—C2	−178.85 (18)
N1^i^—Cu1—N1—C2	−37.46 (15)	C3—C2—C7—C6	−1.3 (3)
Cl1^i^—Cu1—N1—C2	−171.56 (17)	N1—C2—C7—C6	178.54 (18)
Cl1—Cu1—N1—C2	73.71 (19)	C3—C2—C7—N2	−179.11 (18)
C2—N1—C1—N2	0.9 (2)	N1—C2—C7—N2	0.7 (2)
Cu1—N1—C1—N2	174.20 (13)	C1—N2—C8—C9	−134.1 (2)
C7—N2—C1—N1	−0.4 (2)	C7—N2—C8—C9	44.3 (3)
C8—N2—C1—N1	178.25 (18)	C1—N2—C8—C13	45.5 (3)
C1—N1—C2—C3	178.8 (2)	C7—N2—C8—C13	−136.1 (2)
Cu1—N1—C2—C3	6.4 (3)	C13—C8—C9—C10	1.5 (3)
C1—N1—C2—C7	−1.0 (2)	N2—C8—C9—C10	−178.90 (19)
Cu1—N1—C2—C7	−173.47 (14)	C8—C9—C10—C11	−0.7 (3)
C7—C2—C3—C4	1.4 (3)	C9—C10—C11—C12	−1.2 (3)
N1—C2—C3—C4	−178.4 (2)	C9—C10—C11—C11^ii^	178.2 (2)
C2—C3—C4—C5	−0.4 (3)	C10—C11—C12—C13	2.4 (3)
C3—C4—C5—C6	−0.8 (3)	C11^ii^—C11—C12—C13	−176.9 (2)
C4—C5—C6—C7	0.9 (3)	C11—C12—C13—C8	−1.8 (3)
C5—C6—C7—N2	177.3 (2)	C9—C8—C13—C12	−0.2 (3)
C5—C6—C7—C2	0.2 (3)	N2—C8—C13—C12	−179.86 (18)
C1—N2—C7—C6	−177.7 (2)		

Symmetry codes: (i) −*x*+2, *y*, −*z*+3/2; (ii) −*x*+1, *y*, −*z*+5/2.
